# A Review of the Literature on Primary Leiomyosarcoma of the Prostate Gland

**DOI:** 10.1155/2015/485786

**Published:** 2015-11-12

**Authors:** Anthony Kodzo-Grey Venyo

**Affiliations:** Department of Urology, North Manchester General Hospital, Delaunays Road, Manchester, UK

## Abstract

Primary leiomyosarcoma of the prostate (PLSOP) is rare, with less than 200 cases reported so far. PLSOPs present with lower urinary tract symptoms, haematuria, and perineal pain; may or may not be associated with a history of previous treatment for adenocarcinoma of prostate by means of radiotherapy and or hormonal treatment; may afflict children and adult male. Examination may reveal benign enlarged prostate and hard enlarged mass. PLSOPs may be diagnosed by histological examination findings of spindle-shaped carcinoma cells in prostate specimens. Immunohistochemical staining tends to be positive for vimentin, CD44, smooth muscle actin, and calponin, focally positive for desmin, and at times positive for keratin. They stain negatively for PSA, S-100, CD34, CD117, and cytokeratin. Cytogenetic study on primary leiomyosarcoma of the prostate gland may show clonal chromosomal rearrangement involving Chromosomes 2, 3, 9, 11, and 19. On the whole the prognosis is poor. Surgery with or without chemotherapy would appear to be the mainstay of treatment for PLSOPs that are operable, but generally there is no consensus opinion on the best therapeutic approach. Most cases of PLSOPs are diagnosed in an advanced stage of the disease. A global multicenter trial is required to find therapies that would improve the prognosis.

## 1. Introduction

Sarcoma of the prostate gland is rare and this accounts for only 0.1% of all malignant tumours of the prostate gland [1]. Leiomyosarcoma of the prostate gland is rare and globally less than 200 cases have been reported in the literature and less than 100 PLSOPs have been reported in the English literature [2]. Leiomyosarcoma accounts for approximately 25% of all sarcomas of the prostate gland. [3] Leiomyosarcoma of the prostate was first described in 1853 by Sambert as stated by Riba et al. [4] in 1950. Leiomyosarcoma of the prostate gland is an aggressive tumour which most clinicians globally have not encountered; in view of this most clinicians would not be familiar with its biological behaviour. The ensuing literature review on primary leiomyosarcoma of the prostate gland is divided into two parts: (A) an overview and (B) miscellaneous narrations and discussions from some reported cases of leiomyosarcoma of the prostate gland.

## 2. Methods

Various internet data bases were searched, including Google, Google Scholar, PubMed, and Educus, for information on primary leiomyosarcoma of the prostate gland. The search terms used included primary leiomyosarcoma of the prostate gland; leiomyosarcoma of the prostate gland; leiomyosarcoma of prostate; prostatic leiomyosarcoma; primary prostatic leiomyosarcoma. Fifty-five references related to case reports, case series, and other types of the literature related to primary leiomyosarcoma of the prostate gland were identified suitable for writing the literature review.

## 3. Literature Review

*(A) Overview*

*General Comments*

 Primary leiomyosarcoma of the prostate gland (PLSOP) is the commonest sarcoma affecting the prostate gland, even though on the whole it is a rare tumour [5].
 PLSOPs cause obstruction and tend to involve adjacent organs [5].
 PLSOPs tend to recur [5].
 PLSOP metastasizes to the liver and lung [5].
 PLSOP tends to be associated with a mean survival of 3 to 4 years [5].

*Definition*. Sarcoma of the prostate is a rare type of malignancy of mesenchymal origin that can rarely afflict the prostate gland. Various types of sarcoma of the prostate gland can occur including leiomyosarcoma, which is a smooth muscle sarcoma, and rhabdomyosarcoma, a skeletal muscle-type sarcoma. 

*Aetiology of Sarcoma of the Prostate Gland*. On the whole the aetiology of PLSOP is unknown or not well understood. 

*Sarcomatous Dedifferentiation [6]*. A number of cases of leiomyosarcoma of the prostate gland develop a number of years after patients had undergone treatment by means of radiotherapy and/or hormonal treatment for adenocarcinoma of the prostate gland. Even though there may be absence of any residual adenocarcinoma of the prostate, evidence of a new malignancy in the form of leiomyosarcoma of the prostate could be conjectured to be a sequel of dedifferentiation of totipotential cells in the prostate gland as an emanation from previous effect of the radiotherapy or hormonal therapy on the totipotential cells. 

*Li-Fraumeni Syndrome [6–8]*. Li-Fraumeni syndrome has been postulated to be associated with mutation of p53 tumour suppressor gene and this leads in some children to the development of sarcomas and their mothers tend to have an increased risk of carcinoma of the breast with siblings having an increased risk for the development of malignancy [7, 8].

*Age Distribution*. Leiomyosarcoma of the prostate gland has been reported in children and adults whose ages have ranged from 2.5 years to 80 years (see the case of the 2.5-year-old boy in [9]). 

*Presentation*. Patients with PLSOPs tend to present with the following (see [2, 10, 11] for some of the presentations):

1. “Lower urinary tract symptoms” including urinary frequency, poor flow, hesitancy, urinary urgency, and inability to void. The presentation of lower urinary tract symptoms may mimic benign prostatic hypertrophy clinically in that rectal examination findings may indicate benign prostatic hypertrophy and the diagnosis is established after the patient has undergone transurethral resection of prostate and the histology result would indicate leiomyosarcoma of the prostate.
2. Haematuria: sometimes a history of previous treatment for adenocarcinoma of prostate by means of hormonal therapy and/or radiotherapy in the form of external beam radiotherapy or brachytherapy may be obtained
3. Retention of urine may occur.
4. They may present with an exophytic mass of prostate projecting towards or infiltrating the rectum or a perineal mass.
5. Leiomyosarcomas of the prostate may manifest with metastases to the liver and lung [12].

*Clinical Findings*. Digital rectal examination may reveal the following:

1. An enlarged prostate which may feel benign.
2. An enlarged/firm prostate gland.
3. A hard prostate which has extended to the capsule or distorted the capsule.
4. An enlarged hard mass arising from the prostate and extending to the rectum, pelvic side wall, perineum, and seminal vesicle or involving the base of the urinary bladder.
5. An enlarged mass arising in the prostate gland in a child with voiding symptoms.

*Laboratory Investigations*. The following investigations tend to be undertaken in cases of PLSOP:

1. Urine: urinalysis may be normal or may show evidence of visible or nonvisible haematuria and these are not specific for a diagnosis of leiomyosarcoma of the prostate but form part of the general assessment of the patient. Urine microscopy and culture may or may not show evidence of infection and this examination is used as part of the general assessment of the patient.
2. Blood tests: routine blood tests are undertaken in the assessment of the patient including full blood count, serum urea and electrolytes, liver function tests, serum glucose, and coagulation screen. If the patient is anaemic, then the appropriate management is undertaken to correct the anaemia. Furthermore attempts tend to be made to correct any impairment noticed in the blood test results to improve upon the general management of the patient. Serum PSA may be normal or raised, but this would not be diagnostic of leiomyosarcoma of the prostate and the level of serum PSA would not be helpful to determine the progress of leiomyosarcoma of the prostate following treatment (see [13], e.g., when serum PSA was normal).

*Radiological Investigations*. Radiological investigations which may be undertaken in PLSOP include the following:

1. Ultrasound scan (see [14] when ultrasound scan of abdomen and pelvis was done): ultrasound scan of the abdomen and pelvis would assess whether or not the patient has hydronephrosis; it can also indicate whether or not there is thickening of the bladder wall or trabeculation or sacculation or if there is a blood clot in the urinary bladder. Infiltration of the base of the bladder or any part of the bladder can be illustrated by ultrasound scan. Enlargement of the prostate as well as the characteristic features of the prostate gland and its relationship to nearby structures can be illustrated by ultrasound scan.
2. Transrectal ultrasound scan of prostate (TRUSP) and biopsies (see [10, 15] in which transrectal ultrasound scan of prostate and biopsies were undertaken): transrectal ultrasound scan of prostate is a useful technique for the assessment of the characteristics of the prostate gland which may show enlargement of the whole or part of the prostate gland involved. It may show heterogeneous hypoechoic lesions in the prostate, invasion of the capsule or extension into the rectum, pelvic side wall, seminal vesicle, ejaculatory duct, or protrusion into the urinary bladder or infiltration of the wall of the urinary bladder. Transrectal ultrasound-guided biopsy tends to be the usual approach for obtaining specimens of the prostatic lesion for histological examination which would establish the diagnosis of leiomyosarcoma of the prostate. A patient who had previously undergone radiotherapy by means of external beam method or brachytherapy or who has been having hormonal treatment for adenocarcinoma of prostate gland upon subsequent development of relapse and further enlargement of prostate may undergo a further ultrasound-guided biopsy of the prostate gland and its histological examination on the second occasion may reveal leiomyosarcoma of the prostate.
3. Computed tomography (CT) scan (see [10, 11, 13] in which CT scan was used): CT scan of abdomen, pelvis, and thorax can be used to assess the features of the prostatic lesion, the relation of the lesion to nearby structures, and the extent of the disease locally. It can also show whether or not there is lymphadenopathy or metastases in the abdomen or within the thorax. CT scan can also be used in the follow-up assessment of the patient following treatment.
4. Magnetic resonance imaging (MRI scan) (see [15] in which MRI scan was used): MRI scan of abdomen, pelvis, and thorax can be used to assess the features of the prostatic lesion, the relation of the lesion to nearby structures, and the extent of the disease locally. It can also show whether or not there is lymphadenopathy or metastases in the abdomen or within the thorax. MRI scan can also be used in the follow-up assessment of the patient following treatment.
5. Isotope bone scan (see [10] in which isotope bone scan was used): isotope bone scan can be used in the initial as well as follow-up assessment of the patient to ascertain whether or not there is bone metastasis.

*Cystoscopy Findings*

- Cystoscopy may be undertaken in the assessment of the patient and this may show an enlarged prostate gland. Cystoscopy may at times show enlarged necrotic or bleeding median lobe of the prostate; it may also show protrusion or invasion of the prostatic lesion into the urinary bladder as well as trabeculation of the urinary bladder or blood clot in the urinary bladder. Nevertheless, these findings are not specific to leiomyosarcoma of the prostate gland.
- Transurethral resection biopsy of a bleeding or necrotic median lobe can be undertaken after cystoscopy for histological examination which may lead to a diagnosis of leiomyosarcoma of the prostate.
- At the end of cystoscopy transurethral resection of the prostate gland may be done for a presumed benign prostatic hypertrophy requiring resection to improve urinary flow or to relieve urinary retention and the histological examination may incidentally reveal leiomyosarcoma of the prostate gland.

*Macroscopic Features*

- Leiomyosarcomas of the prostate tend to be large tumours arising from the prostate and breaching the capsule of the prostate, extending into the perineum, towards the side wall of the pelvis or projecting into or invading the urinary bladder. When the median lobe is involved necrotic areas and bleeding areas may be seen of the median lobe of the prostate. The macroscopic features of leiomyosarcoma of the prostate gland are not specific features.
- The size of PLSOPs tends to vary from 3 cm to 21 cm and infiltrative [2].
- Macroscopic examination of PLSOPs tends to reveal a non-well-defined mass which may be fleshy with areas of necrosis and bleeding.

*Microscopic Features*

- Microscopic examination of leiomyosarcoma of the prostate tends to reveal spindle cells with enlarged hyperchromatic nuclei with evidence of increased mitotic activity [10].
- Most cases of PLSOP tend to have high-grade features on microscopic examination associated with areas of viable tumour which comprise hypercellular, intersecting bundles of eosinophilic, spindle-shaped cells that have variable degrees of nuclear mitotic activity as well as nuclear atypia. Some cases may have low-grade features [13].

*Immunohistochemistry*

*Positive Immunohistochemistry*. Leiomyosarcomas of the prostate tend to stain positively for the following:

- Vimentin [2, 13].
- CD44 [13].
- Smooth muscle actin—about 63% may stain positive for actin [2].
- Calponin—focally positive [11].
- Desmin—20% of cases may stain weakly for desmin [2].
- Keratin—27% of cases may stain positive for keratin [2].

*Negative Immunohistochemistry*. Leiomyosarcomas of the prostate gland tend to stain negatively for the following:

- S-100 [13].
- Cytokeratin [13].
- CD117 (c-kit) [13].
- PSA [11].
- CD34 [11].

*Cytogenetics/Molecular Genetic Tests*. Cytogenetic study on primary leiomyosarcoma of the prostate gland may show clonal chromosomal rearrangement involving Chromosomes 2, 3, 9, 11, and 19 [16]. 

*Electron Microscopic Features*. Electron microscopic examination of specimens of leiomyosarcoma of the prostate gland may show the bulk of the cytoplasm to be filled with microfilaments which are arranged on a long axis [15]. 

*Differential Diagnoses*. The differential diagnoses of PLSOP include the following:

- Nodular hyperplasia of prostate with atypical changes: in this case microscopic examination would reveal no evidence of invasion and no evidence of mitotic figures [2]. In nodular hyperplasia of the prostate gland microscopic examination of the specimen of the prostate tends to show hyperplasia of stroma and glandular tissue with evidence of papillary buds; in-folding and cysts, associated with squamous metaplasia and necrosis; the microscopic features which tend to begin around the urethra where the ejaculatory ducts enter the prostate gland (in the transitional and periurethral zone); the basal layer which tends to be continuous, evidence of stromal changes including increased smooth muscle, lymphocytes, and ducts which in the majority of cases are not associated with infectious process of prostatitis, reduced elastic tissue; various other features including sclerosing adenosis, fibroadenoma-like and phyllodes-like hyperplasia, leiomyoma-like nodules, and fibromyxoid nodules associated with infarct [17]. Nodular hyperplasia of prostate gland on immunohistochemistry tends to be positive for CD10 [18]. It has been stated that in nodular hyperplasia of the prostate gland no well-organized fascicles are seen on microscopic examination of the prostatic lesion and also there is hyalinization, no necrosis, and no calcification on microscopic examination [12].
- Postoperative spindle cell nodules: diagnosis of this condition could be based upon history of previous prostatic operation and microscopic examination tends to reveal no evidence of invasion and no evidence of mitotic figures [2]. Microscopic examination of the prostate specimen involved with postoperative spindle cell nodule tends to show evidence of cellular tissue with high mitotic activity, interlacing fascicles of spindle cells with extravasation of red blood cells which resemble Kaposi's sarcoma, minimal nuclear pleomorphism, and no atypical mitosis, and the size of the lesion tends to be relatively small [19].
- Gastrointestinal stromal tumour of rectum (Rectal GIST): it has been stated that there may be difficulties differentiating between PLSOP and Rectal GIST [20]. In Rectal GIST microscopic examination tends to show transmural, usually plump, spindle cells with eosinophilic cytoplasm within stroma that tend to be variably hyalinised or oedematous, skeinoid fibres which are extracellular globules tend to be commonly seen, muscle infiltration may also be seen, there may be evidence of epithelioid morphology, and one to two mitotic figures may be seen per ten high-power fields [21]; on rare occasions there may be evidence of osteoclast-like giant cells [21, 22]. In Rectal GIST immunohistochemistry tends to reveal positive staining with CD117, CD34, and vimentin; immunohistochemistry also tends to show positive staining in 30% to 40% of cases for alpha smooth muscle actin and in 5% of cases for S100. Variable weak positive staining for keratin may also be seen in Rectal GIST [21]. In Rectal GIST electron microscopic examination of the specimen tends to show predominantly features of smooth muscle differentiation including long interdigitating cytoplasmic processes, intercellular junctions, and dense core granules [21]. In Rectal GIST electron microscopic examination also tends to show features of predominant neural differentiation including neuron-like cells which have axonal cytoplasmic processes, synapse-like structures, and dense core neurosecretory granules [21].
- Some PLSOPs mimic stromal sarcoma of the prostate and sarcomatoid carcinoma of the prostate. Macroscopic examination of stromal sarcoma of the prostate specimen may reveal a whitish-yellow multinodular appearance with focal necrosis; microscopic examination of the specimen may show sarcomatoid oval to spindle cells; immunohistochemical staining of the specimen tends to show the membrane of the tumour cells to be positive with CD56, focal positivity in the cytoplasm of the tumour cells for synaptophysin, and positive staining on the cell membrane of the tumour cells for CD99 [23]. Microscopic examination of sarcomatoid carcinoma of the prostate gland (carcinosarcoma of the prostate) tends to reveal a biphasic tumour with adenocarcinoma and recognizable sarcoma components including chondrosarcoma, rhabdomyosarcoma, angiosarcoma, osteosarcoma, and leiomyosarcoma [24]. Immunohistochemistry of sarcomatoid carcinomas of the prostate tends to exhibit positive staining for the epithelial component for cytokeratin and PAP but negative staining for PSA [25]. Immunohistochemistry of the sarcoma component of sarcomatoid carcinoma of the prostate tends to reveal negative staining for PSA, EMA, and keratin [24].
- Other PLSOPs may mimic leiomyomas. In leiomyomas of the prostate gland microscopic examination of the prostate tends to show well-organized fascicles of spindle cells [12]. In leiomyoma of the prostate the lesion tends to be normocellular with no or rare mitotic activity and no or rare nuclear atypia, but there may be evidence of atypical bizarre stromal cells [26, 27]. Leiomyomas of the prostate gland tend to stain positively with desmin, actin, and androgen receptor on immunohistochemical staining [12]. On the other hand, leiomyosarcomas of the prostate gland microscopic examination of the prostatic lesion tend to show hypercellularity, evidence of infiltration, variable atypia, definite mitotic activity, and necrosis [12].

*Treatment*. Treatment options that can be undertaken in cases of leiomyosarcoma of the prostate gland include the following:

1. Radical surgery in the form of radical prostatectomy or exenteration alone or plus or minus chemotherapy alone or plus radiotherapy (see, e.g., [15, 28]).
2. Chemotherapy and radiotherapy (see, e.g., [29]).
3. Chemotherapy alone (see, e.g., [10]).
4. Radiotherapy alone (see, e.g., [13]).
5. Palliative care including pain control and overall supportive care by a multidisciplinary team approach.
6. Patients who have stomas in the form of ileal conduit or colostomies in cases where the rectum is involved and colostomy performed would require support from the stoma nurse.

*Outcome*. The outcome of the disease may be summarized as follows:

1. On the whole the prognosis of leiomyosarcoma of the prostate is poor, but variable survival rates have been reported (see [2]), but it has been stated that the best survival occurs following curative surgery without evidence of residual or metastatic disease. It would appear that curative surgery together with multimodal therapy with chemotherapy would be the best form of treatment if the patient is fit to undergo multimodal treatment, but this approach may be hampered by the advanced stage of the disease at presentation and the general condition of the patients who may not be fit for multimodal treatment.
2. There is no consensus opinion on the best therapeutic approach in view of the rarity of leiomyosarcoma of the prostate; therefore there is need for a global multicentre trial on management of leiomyosarcoma of the prostate in order to form an opinion on the best treatment option that would improve the prognosis.
3. Perhaps if leiomyosarcoma of the prostate is diagnosed at an early stage, then the prognosis may improve following treatment. It may be that if patients who had previously undergone radiotherapy and/or hormonal therapy for adenocarcinoma of prostate are made to undergo prostate biopsies early when there is evidence of relapse or recurrence of their prostate carcinomas those whose tumours may have dedifferentiated into leiomyosarcoma of the prostate would be detected early, but this is conjectural.
4. Leiomyosarcomas of the prostate gland are aggressive tumours with a median survival of 3 to 4 years and they tend to recur as well as metastasize to the liver and the lungs [5].

*(B) Miscellaneous Narrations and Discussions from Some Reported Cases (See Table 1 for a List of Some of the Reported Cases and Case Series)*. Cheville et al. [2] undertook a clinicopathological study of all of the cases of leiomyosarcoma of prostate that had been managed in their institution from 1929 to 1994. They had retrieved twenty-three cases from their files and out of these clinical follow-up data were available for 14 patients. Immunohistochemical studies had been undertaken including actin, desmin, S-100 protein, keratin, and vimentin. With regard to the results, Cheville et al. [2] reported the following:

- The ages of the patient had ranged between 41 years and 78 years and their mean age was 61 years.
- All of the patients (100%) presented with urinary obstruction. Twenty-five percent of the patients presented with perineal pain. Seven percent of the patients had presented with burning on ejaculation and seven percent of the patients had presented with weight loss.
- The dimensions of the prostatic tumours had ranged from 3.3 cm to 21 cm in the greatest dimension with a mean dimension of 9 cm.
- The tumours quite often were associated with necrosis.
- With regard to the histological grading of the tumours 7 tumours were assigned grade 2; 10 tumours were assigned grade 3 and 6 tumours were assigned grade 4. The grading of the tumours was based upon Broder's grading system (scale, 1–4).
- The mitotic figure counts had varied between 2 and 24 per 10 high-power fields.
- With regard to immunohistochemistry, fifteen of 15 (100%) of the tumours were immunohistochemically positively stained for vimentin, 10 out of 16 (63%) were positively stained for actin, and 3 out of 15 (20%) were weakly reactive for desmin. Immunohistochemical expression was observed for keratin in 15 (27%) of the cases and, furthermore, all of the tumours (100%) were negatively stained for S-100.
- With regard to treatment, the patients underwent various types of treatment which usually included a combination of radiotherapy, chemotherapy, and radical prostatectomy or cystoprostatectomy.
- The follow-up of the patients had ranged from 2 months to 72 months and the mean follow-up was 19 months.
- With regard to the outcome of the patients, 10 patients had died 3 months to 72 months (mean, 22 months) pursuant to their diagnosis and 4 patients were alive at the time of publication of the paper which included three patients who had residual tumour and one patient who did not have any evidence of tumour at 1 month, 4 months, 30 months, and 4.5 months, respectively. Ten out of 11 patients had developed local recurrence which included 5 patients who had gross residual tumour present after their surgery. Metastases had developed up to 40 months pursuant to their surgery (with a mean time of 10.3 months after their surgeries) and most of the metastases had involved the lungs.

Cheville et al. [2] made the ensuing conclusions:

- Their findings indicated that leiomyosarcoma of the prostate gland has a variable histological appearance which ranges from spindle cell tumours reminiscent of smooth muscle to pleomorphic sarcoma.
- Epithelioid characteristics may be present in the tumours.
- The majority of the tumours on immunohistochemical staining were positively stained with vimentin and actin and immunoreactivity with antikeratin antibodies would not exclude the diagnosis of leiomyosarcoma.
- Leiomyosarcoma of the prostate gland has a poor prognosis, even though the duration of survival has been variable.
- Radical surgery had been the treatment modality of choice in their series, but it was difficult to achieve complete excision of tumour in the majority of cases and the surgical operations did not result in cure.

**Figure 1:**
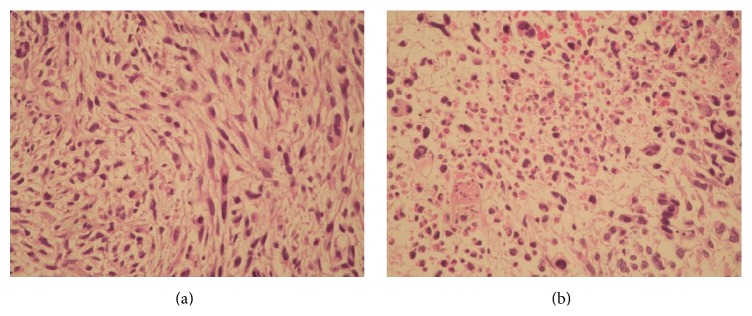
(a) Leiomyosarcoma composed of a dominant population of neoplastic spindle cells: (a) intermingled with giant neoplastic cells and multifocal and multifocal necrosis (b). Reproduced from [13].

**Figure 2:**
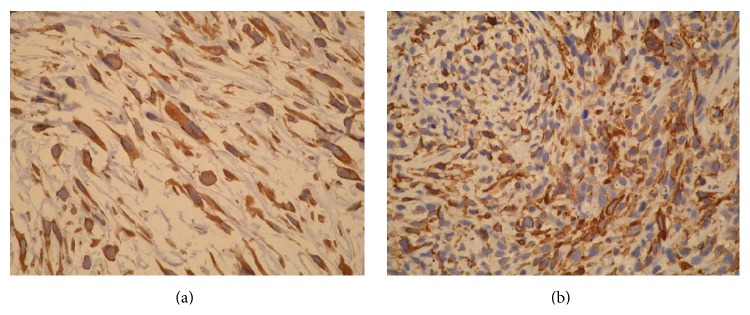
(a) and (b) Immunohistochemistry demonstrates that tumour cells express smooth muscle actin (a) and vimentin (b). Reproduced from [13].

Vandoros et al. [13] reported an 80-year-old man who had presented with urinary frequency, dysuria, poor urinary stream, and nocturia. His rectal examination showed a firm nodular mass of 3 cm to 4 cm diameter which had involved the left lobe of the prostate gland and which had extended to the edge of the gland. The right lobe of the prostate gland was noted to be diffusely firm. His serum prostate specific antigen (PSA) level at presentation was 2.7 ng/mL and this had not changed over 3 years. He underwent transurethral resection of prostate (TURP) and histological examination of the specimen showed a dominant population neoplastic spindle cells which were intermingled with giant neoplastic cells and multifocal necrosis which had involved almost the entire tumour (see Figures 1(a) and 1(b)). Immunohistochemical staining of the tumour was reported to have confirmed the diagnosis of leiomyosarcoma of the prostate gland in that the tumour cells on immunohistochemistry expressed positive staining with smooth muscle actin (see Figure 2(a)), vimentin (see Figure 2(b)), and CD44, and the tumours on immunohistochemistry exhibited negative staining for S-100, cytokeratins, and CD117 (c-KIT). He had computed tomography (CT) scan of the abdomen and thorax which had revealed two hypodense liver lesions, multiple pulmonary nodules, and mediastinal and left hilar lymphadenopathy, which were all considered to be suspicious of metastases. He also had CT scan of the brain which did not show any metastasis. He did not want any intervention; therefore, he was treated symptomatically. Three months subsequently, he presented with retention of urine and acute renal failure for which a long-term urinary catheter was inserted and for which palliative external beam radiotherapy was recommended, but he refused to undergo radiotherapy, so he was discharged home with hospice care. He died 2 months later. Vandoros et al. [13] stated that they had reviewed 54 cases of leiomyosarcoma which had been reported prior to the publication of their case and their review had revealed the following:

- The mean survival of leiomyosarcoma of the prostate gland was estimated at 17 months (95% CI, 20.7–43.7 months).
- The 1-, 3-, and 5-year actuarial survival rates were 68%, 34%, and 26%, respectively.
- The only factors predictive of long-term survival were negative surgical margins and absence of metastatic disease at the time of initial presentation.

Vandoros et al. [13] concluded that a multidisciplinary approach to the management of leiomyosarcoma of the prostate gland is necessary for the appropriate management of this dire (aggressive) disease.

Singh et al. [10] reported a 35-year-old man who had presented with lower urinary tract symptoms and progressive perineal pain. He was found on examination to have a palpable urinary bladder and a nontender, lobulated asymmetrically enlarged, prostate gland of variegated consistency. His serum PSA level was slightly raised at 5.31 ng/mL. His urine analysis and the rest of his serum biochemical analyses were normal. He had transrectal ultrasound scan of prostate which revealed an enlarged prostate gland with a heterogeneous mass on the left posterolateral aspect of the prostate gland. He had transrectal ultrasound-guided biopsies of the prostate gland and histological examination of the specimens had revealed the tumour to be composed of spindle-shaped cells, with elongated, plump nuclei, nuclear pleomorphism, and brisk bizarre multipolar mitotic activity. A diagnosis of leiomyosarcoma of the prostate gland was made. He had a CT scan of abdomen, pelvis, and thorax which had shown a heterogeneous mass arising from the left posterolateral aspect of the prostate gland with an area of infiltration in the base of the urinary bladder, anterior wall of the rectum, and left posterolateral wall of the pelvis. The CT scan also showed multiple enlarged presacral lymph nodes and small nodules on the base of the lung on the left side. He had isotope bone scan which was normal with no evidence of bone metastasis. The patient received two cycles of combination chemotherapy which consisted of ifosfamide (1600 mg/m^2^) and epirubicin at 40 mg/m^2^ for three days at three-weekly intervals. However, he died one month after receiving his second cycle of chemotherapy.

Dubey et al. [11] reported a 73-year-old man who presented with memory loss. His wife reported that he had been having problems with his bowel movements and at times he had had episodes of severe rectal burning. He had also been having lower urinary tract symptoms. He had colonoscopy which was on the whole normal but which showed a benign polyp at 35 cm. His serum biochemistry tests were normal except for evidence of hyponatraemia which was considered to be the reason for his confusion. His serum PSA at initial manifestation was 2.7 ng/mL and this had not changed over 3 years. He had a CT scan of brain, thorax abdomen, and pelvis which had shown a very large nodular prostate gland of about 6 cm in size and which had projected into the left posterior inferior aspect of the urinary bladder and had indented into the lumen of the rectum and this was adjudged to have almost obstructed the lumen of the rectum at the level of the dentate line. There was no evidence of lymphadenopathy or metastasis. He had a digital rectal examination which had revealed a very large, hard, nodular prostate gland projecting into the lumen of the rectum and almost causing obstruction of the lower gastrointestinal tract. He could not tolerate transrectal ultrasound-guided biopsy of the prostate under local anaesthesia because it was too painful. He underwent trocar needle biopsy of prostate under sedation in the operating theatre. During assessment at the time of the prostate biopsy upon assessment of the prostate gland it was felt that the tumour was far more advanced than was previously envisaged. The histopathological findings of the biopsy specimens showed malignant spindle cell tumour which was adjudged to favour leiomyosarcoma of the prostate gland. Immunohistochemichal staining had shown that the spindle cell proliferation was negatively stained for PSA, S100, pancytokeratin, CD117, and CD34. Immunohistochemical staining had shown that the tumour was strongly positively stained for smooth muscle actin, calponin, and CD44 and focally positive for desmin. His perineal pain deteriorated subsequently and he developed urinary retention which required insertion of suprapubic cystostomy. The case was discussed at a multidisciplinary team meeting which concluded that the lesion was leiomyosarcoma of prostate and the treatment would require surgery which should involve total pelvic exenteration or if the tumour was bulky then neoadjuvant chemotherapy with or without radiotherapy, followed by surgery. His hyponatraemia resolved and his mentation had improved; however, with his low performance status, he was adjudged unsuitable to undergo surgical operation as well as not fit to receive chemotherapy. He developed persistent haematuria for which he was able to tolerate a single high dose 8-gray radiotherapy out of the 30 Gy/10 fractions which was planned. His bleeding had stopped in a few weeks, but he had severe pelvic pain which was not controlled by regular narcotics and he required palliative care and he received methadone but died of his disease after two weeks.

Chen et al. [15] reported a 27-year-old man who had had difficulty with voiding. He developed acute retention of urine and was catheterized and one week later was referred to a different institution. He had rectal examination which revealed a solid mass of approximately 11 cm × 8 cm × 7 cm with a smooth surface and elastic consistency. His serum PSA level was 4.8 ng/mL. But his urine analysis and his blood biochemistry were on the whole normal otherwise. He had transrectal ultrasound scan-guided biopsy of prostate and histological examination of the specimen revealed features consistent with spindle cell sarcoma. He had a chest X-ray which was normal. He also had intravenous urography which had shown an atonic urinary bladder with marked residual urine volume after voiding. He had magnetic resonance imaging (MRI) scan of pelvis which showed a large lesion in the pelvis arising from the prostate gland and which had invaded the urinary bladder. He underwent radical cystoprostatectomy and during the operation it was noted that there was involvement of the rectum by the tumour; therefore low anterior resection was undertaken with segmental resection of the rectum and construction of an ileal conduit. Microscopic examination of the tumour had revealed that the tumour had consisted of spindle cells with enlarged hyperchromatic nuclei as well as increased mitotic activity. Immunohistochemical staining of the tumour had shown positive staining for vimentin and weakly positive staining for actin as well as negative staining for desmin. The tumour was therefore adjudged to have illustrated leiomyosarcoma of the prostate gland with invasion of the urinary bladder and rectum. Furthermore, electron microscopic examination of the tumour revealed features of spindle cell sarcoma which established a definite pathological diagnosis of leiomyosarcoma of the prostate gland. He had remained alive for 48 months at the time of publication of the paper with no evidence of recurrence of his tumour. Lessons learnt from this case would indicate the following: leiomyosarcoma of the prostate gland could be locally advanced, but if radical surgery is undertaken and there is complete clearance of tumour without any evidence of residual disease even without adjuvant chemotherapy or radiotherapy some of the patients would tend to have a medium/long-term survival.

Vakilha et al. [29] reported a 72-year-old man who presented with urinary dribbling, hesitancy, and perineal pain. He had rectal examination which revealed a firm and immobile prostatic mass. He had CT scan which showed a nonhomogeneous prostatic mass which was confined to the prostate gland and not invading other organs and it also showed no evidence of lymph node involvement. He had CT scan of thorax and chest X-ray which were normal. His serum PSA level at presentation was 4.2 ng/mL. He had transrectal ultrasound-guided biopsy of prostate and histological examination of the specimen revealed features which were considered to be suspicious of malignancy and the patient underwent radical surgery (prostatectomy). Histological examination of the specimen showed a high-grade spindle cell sarcoma which was diagnosed as leiomyosarcoma of prostate with clear surgical margin. Immunohistological examination of the tumour showed that the tumour cells were weakly stained with desmin, but EMA staining was negative. This result was adjudged to be consistent with a diagnosis of leiomyosarcoma of prostate gland. He initially received one course of chemotherapy with three drug regimens which consisted of ifosfamide, dacarbazine, and farmorubicin. He did not want to continue having chemotherapy. He, therefore, received 4500 cGy of radiotherapy to the pelvis and 1500 cGy to the prostatic bed. He was initially well for 10 months; nevertheless, he represented with shortness of breath and he had CT scan of thorax which showed multiple lung metastases. He had a rectal examination which revealed a nontender firm nodule. He was started on chemotherapy with the MAID regimen (mesna, Adriamycin, ifosfamide, and dacarbazine). After he had received four courses of chemotherapy, a partial response was observed both in his radiological image findings and with regard to his symptoms. He was still receiving chemotherapy at the time of publication of the paper.

Horiguchi et al. [28] reported a 69-year-old man who had been diagnosed as having adenocarcinoma of prostate gland and who had been treated by means of brachytherapy. Six years after he had undergone brachytherapy, he developed dysuria and visible haematuria. He underwent urethroscopy which had shown a stenosis caused by a tumour at the level of the prostate. He underwent transurethral resection of the prostate and histological examination of the tumour showed features consistent with a diagnosis of leiomyosarcoma of the prostate gland. He was treated by means of three cycles of neoadjuvant chemotherapy which consisted of doxorubicin and ifosfamide. And this was followed by radical cystoprostatectomy and pelvic lymphadenectomy. It was found that the tumour originated from the prostate gland and had infiltrated the wall of the urinary bladder and serosa with lymphatic and venous invasion. The surgical margin was clear of tumour and there was no evidence of residual adenocarcinoma of the prostate gland on histological examination. Histological examination had also revealed that the proportion of necrotic tumour cells which had been induced by the chemotherapy was 50%. He was offered adjuvant chemotherapy later on, but he opted to be followed up without receiving any further chemotherapy. At three months pursuant to his surgical operation, he had a CT scan of abdomen, pelvis, and thorax which showed local recurrence and lung metastasis. He was then treated with ifosfamide; nevertheless, he did not respond to chemotherapy and he died six months after the operation. Horiguchi et al. [28] concluded that effective treatment strategy for sarcoma of the prostate gland should be developed in the near future, although the clinical features of sarcoma of the prostate gland remain unclear due to its rare incidence. The development of dedifferentiation of totipotential cells in the prostate gland following radiotherapy to the prostate gland or hormonal therapy for adenocarcinoma has been known.

Sastri et al. [14] reported a 60-year-old man who presented with dysuria, lower abdominal pain, and pain in his left lower limb. He had undergone open prostatectomy in another hospital from where he was referred. On examination he was found to have on palpation underneath his hypogastric surgical scar a 5 cm diameter, almost globular nontender, immobile mass. He had a rectal examination which revealed a large, hard growth anterior to his rectum with obliteration of the median sulcus of the prostate gland and the upper border of the prostate gland could not be reached. He had ultrasound scan of the abdomen and pelvis and this revealed a mass which measured 104 mm × 94 mm in the region of the prostate gland. He had transrectal ultrasound scan of the prostate which showed the mass to be predominantly echogenic in the central part of the prostate with compression of the peripheral zone. A provisional diagnosis of benign prostatic hyperplasia was made. His serum PSA was 3 ng/mL. He later on had open biopsy of the prostate gland and histological examination of the specimen showed diffusely infiltrating spindle cell tumour which was arranged in interlacing fascicles. There was also evidence of moderate to marked nuclear pleomorphism and high mitotic activity. But there was no evidence of epithelial elements. Immunohistochemical staining of the tumours showed positive staining for desmin and smooth muscle actin; however, immunohistochemistry was negative for cytokeratin and PSA. A diagnosis of primary leiomyosarcoma of the prostate gland was made. He received 30 Gy of palliative external beam radiotherapy in 10 fractions over two weeks. The patient was doing well at his six-month follow-up. This case had illustrated the use of external beam radiotherapy in the palliative treatment of primary leiomyosarcoma of prostate, but considering the fact that the case was reported after 6-month follow-up one cannot predict the medium-term and long-term outcome of the patient with regard to the effect of the radiotherapy alone as treatment.

Germiyanoglu et al. [9] reported a 2.5-year-old boy who had presented with difficulty in voiding. He had had urinary frequency for 7 days prior to the onset of his voiding difficulty. He had a rectal examination which revealed a mass which measured 6 cm × 4 cm × 2 cm in the prostatic fossa. His urine examination and blood biochemistry results were normal except for his serum urea nitrogen which was raised at 40 mg/dL. He had an X-ray of the chest which was normal. He also had a cystogram which had shown that the urinary bladder had been displaced superiorly. He had computed tomography (CT) scan which had shown a 51 mm diameter hypodense homogeneous mass within the location of the prostate gland. He had transrectal ultrasound-guided biopsy of the prostate gland and histological examination of the specimen had shown leiomyosarcoma of the prostate gland. The family of the patient did not accept the offer of radical surgical operation and in view of this a suprapubic cystostomy was carried out and chemotherapy and radiotherapy were planned. After the initial days of receiving chemotherapy, the patient was not brought back to complete the planned treatment. He died 4 months later. Germiyanoglu et al. [9] stated the following: Mottola et al. [50] had postulated that chronic inflammatory processes are responsible for causing leiomyosarcoma of the prostate gland in view of the fact that it had been known that hyperplasia had been observed in the smooth muscles of the prostate gland in chronic prostatitis; nevertheless, the reason behind the aetiology of leiomyosarcoma of the prostate has not been clarified; Palma et al. [39] had stated that leiomyosarcoma reaches a peak in childhood; however, leiomyosarcoma of the prostate gland is most often encountered in the 6th decade; the commonest manifestations tend to be symptoms associated with urinary obstruction and these include dysuria, nocturia, urinary frequency, or retention of urine; Barone and Joelson [36] had stated that leiomyosarcoma of the prostate gland should be differentiated from benign prostatic hyperplasia, carcinoma of the prostate gland, prostatic abscess, mullerian duct cysts, and retrovesical tumours [36].

Yee et al. [37] reported a 75-year-old man who presented with haematuria. He had rectal examination which had revealed a mass in the prostate. He had a history of having been diagnosed 8 years earlier with a stage T2b adenocarcinoma of the prostate gland which was treated by means of external beam radiotherapy and brachytherapy. Both his treatments had failed in that his serum prostate specific antigen (PSA) had been increasing. Four years subsequently he had undergone cryosurgical ablation for a poorly differentiated Gleason 4 + 5 = 9, adenocarcinoma of the prostate gland which was clinically staged as T2b. He had isotope bone scan and CT scan of abdomen and pelvis which showed absence of metastasis. His serum PSA prior to his cryosurgical ablation treatment was 11.4 *μ*g/L and his prostate volume was 26 cc and his rectal examination had revealed a small irregular prostate with induration over both lobes of the prostate. Pursuant to his cryotherapy his serum PSA had decreased to 0.2. After the cryotherapy his serum PSA had increased to 1.5 after one year and 2.7 after two years and he was found to have a questionable induration at the base of the prostate. He had a CT scan which had shown a 2.3 cm mass anterior to the rectum behind the seminal vesicles. He was commenced on hormonal therapy and he received bicalutamide 50 mg orally daily and 30 mg leuprolide depot injections every 4 months and after 8 months his serum PSA had dropped to 0.1. Rectal examination then had revealed a small benign feeling prostate gland. The serum PSA had remained at 0.1 whilst he remained on hormonal therapy, but 2 years later he developed visible haematuria at which time his serum PSA had remained at 0.1 and his rectal examination had shown his prostate gland to be indurated and of about 30 grams. He underwent cystoscopy which had revealed an enlarged necrotic and bleeding median lobe of his prostate gland. He had CT scan which had shown an increase in the size of the prostatic neoplasm which had invaded the rectum, but it had not invaded the pelvic side wall and there was no lymph adenopathy. He had prostate biopsies and histological examination of the specimens showed infiltrative, interlacing fascicles of spindle cells which had eosinophilic cytoplasm and had exhibited high cellularity, marked nuclear atypia, and many atypical mitotic figures which were suggestive of sarcoma of the prostate gland. Immunohistochemical staining of the specimen revealed negative staining for PAS, PCA3, CK A1/A3, CK 903, PSA, and hormone receptor ER/PR which had ruled out any residual adenocarcinoma of the prostate gland. Immunohistochemichal stains for leiomyosarcoma were positive in that desmin and smooth muscle actin were weakly positive, and vimentin was strongly positive. He underwent radical cystoprostatectomy and bilateral pelvic lymph adenectomy, resection of sigmoid colon, and rectum, colostomy, and ileal conduit construction. Histopathological examination of the specimen had revealed features consistent with high-grade leiomyosarcoma of the prostate gland. Pathological examination had shown that the tumour had replaced the prostate gland completely and had extended into the periprostatic fat and the urinary bladder. There was evidence of perineural invasion but no evidence of angiolymphatic involvement. The tumour had also extended and involved the full thickness of the rectum. The tumour additionally had involved the posterior margin of the prostate gland and the deep margin of the rectum; therefore additional deep margins were excised which were free of the designated ink margin. The apex of the prostate was also positive for tumour. Pathological examination of the resected rectum had shown infiltration by high-grade leiomyosarcoma which had involved the full thickness of the bowel wall and numerous ulcerations of the bowel mucosa. There was no tumour in the sigmoid colon and the pelvic lymph nodes. Immunohistochemical staining was positive for desmin and smooth muscle actin which supported a diagnosis of leiomyosarcoma. With regard to the differential diagnosis of mesenchymal neoplasms and sarcomatoid carcinoma, these were ruled out by means of immunohistochemical staining which were negative for myogenin, S100, CD34, high molecular weight cytokeratin, and pancytokeratin. His tumour continued to progress and three months later there was evidence of a nodule in the middle lobe of the right lung and a lesion in the area of the prostatic bed as well as rectal stump which was adjudged to represent tumour recurrence and in view of this he received chemotherapy. Several months later he had CT scan which had shown that the tumour near the prostatic bed had increased in size and extended to involve the perineum and abdominal wall and this had resulted in a cutaneous fistula. It had also shown many new pulmonary metastases. Twenty-five months after the surgical operation and chemotherapy he had remained alive with adjudged poor prognosis. Yee et al. [37] stated the following:

- Leiomyosarcoma of the prostate gland is associated with poor prognosis in view of the aggressive biological behaviour of the tumour, lack of early symptoms, and late presentation; the rate of survival varies between 0% and 60% and the survival rates vary from months to years.
- Miedler and MacLennan [51] had stated that 50% to 75% of patients die as a sequel of leiomyosarcoma of the prostate gland after 2.5 years.
- Mondaini et al. [49] had recommended surgical treatment in the form of cystoprostatectomy followed by chemotherapy or radiotherapy for leiomyosarcoma of the prostate gland.
- Surgical operation may give symptomatic relief and may be an option of palliation for patients rather than cure in view of the fact that development of local recurrence and metastasis tends to be common.
- There is no treatment option that has been regarded as optimum; nevertheless, Mansouri et al. [48] had iterated that radical surgery with complete resection of tumour is the therapeutic option which offers the chance of prolonged survival when the tumour has low mitotic activity.
- Dotan et al. [30] had shown that complete surgical resection of tumour can lead to decreased local recurrence and decreased metastasis which prolongs survival. Nevertheless, leiomyosarcomas of the prostate are often diagnosed late in the process of the disease; in view of this the size of the tumour at the time of resection tends to be extensive.
- Sexton et al. [32] did not find any association between survival and negative surgical margins, the tumour size, or stage of the tumour.
- With regard to adjuvant treatment, Sexton et al. [32] and Janet et al. [52] had shown that survival advantage may exist for a combined multimodality therapeutic strategies to improve the outcome of leiomyosarcoma of the prostate gland. However, studies had revealed that uncommon carcinomas which develop pursuant to radiotherapy tend to be aggressive tumours which manifest with metastatic deposits, for which the prognosis tends to be poor irrespective of treatment.
- In view of the fact that sarcomas tend to be associated with a high recurrence rate, it had been recommended that patients with leiomyosarcoma of the prostate gland should be monitored closely with imaging of the chest, abdomen, and pelvis. The reported sites of metastasis in leiomyosarcoma of the prostate gland in order of frequency include the lung, bone, lymph nodes, and brain [53].
- The exact aetiology of leiomyosarcoma of the prostate gland has not been ascertained and there has been an ongoing debate regarding whether radiotherapy to the prostate gland can induce a secondary cancer.
- Moreira et al. [41] had postulated a causal effect of leiomyosarcoma of prostate pursuant to brachytherapy to the prostate gland. Moreira et al. [41] had discussed the complications of brachytherapy, in which 3 patients had developed carcinoma of the prostate gland after they had received brachytherapy. One of the patients had subsequently developed recurrence of adenocarcinoma of the prostate gland, another patient had developed subsequently neuroendocrine tumour of the rectum, and the third patient had subsequently developed leiomyosarcoma of the prostate gland.
- McKenzie et al. [54] had reported three cases of postradiotherapy sarcoma which had developed in the pelvis, 8 years, 15 years, and 16 years, ensuing localized adenocarcinoma of the prostate gland.
- Mazzucchelli et al. [53] had undertaken a study on histological variants of carcinoma of the prostate gland and reported that half of the sarcomatous components (SC) and carcinosarcomas of the prostate gland had developed following hormonal treatment or radiotherapy treatment ensuing an initial diagnosis of acinar adenocarcinoma of the prostate gland. Nevertheless, Mazzucchelli et al. [53] stated that sarcomatous component of carcinosarcoma status of the prostate gland after radiotherapy is not necessarily the only cause of malignancy and that de novo carcinosarcoma of the prostate gland can also develop.
- Prevost et al. [55] had also reported a case of postradiotherapy sarcoma which did develop 8 years after the patient had received extended beam radiotherapy for adenocarcinoma of the prostate gland.

Talapatra et al. [31] reported a 67-year-old man who had presented with a history of recurrent episodes of haematuria and poor urinary stream. He had previously been diagnosed as having had a benign prostatic hypertrophy for which he had undergone transurethral resection of prostate (TURP) tumour elsewhere two years earlier. The histology slides had not been available for review. He had rectal examination which revealed an enlarged, hard prostate gland with obliteration of the median sulcus and the right lobe of the prostate gland was noted to be enlarged and abutting the rectum but not fixed to it. The examination also revealed a mass which had infiltrated the periprostatic tissue and extended to the pelvic side wall. His serum PSA level at presentation was normal. He had ultrasound scan of abdomen and pelvis which revealed a hypoechoic heterogeneous mass within the prostate gland and infiltrating the base of the urinary bladder and invading the lumen of the urinary bladder. The urinary bladder was noted to have a thick wall and it also contained blood clots. He had magnetic resonance imaging (MRI) scan of the pelvis which had revealed a 7.5 cm × 4.3 cm tumour mass in the right lobe of the prostate and which had distorted the capsule and had extended into the periprostatic fat, neurovascular bundle, and the base of the urinary bladder. He underwent cystoscopy which revealed a large fleshy growth in his prostatic urethra and within the lumen of the urinary bladder in association with blood clot in the urinary bladder. The right lobe of the prostate was enlarged especially at the apex. He had biopsy of the tumour mass and histological examination of the specimen had revealed spindle cell sarcoma which had destroyed the prostate gland with only the occasional benign prostatic gland entrapped within the tumour. With regard to the details of the microscopic features of the tumour, the tumour was laid out in intergrating fascicles. The spindle-shaped cells had elongated blunt ended cigar-shaped hyperchromatic nuclei and eosinophilic fibrillary cytoplasm which had the characteristics of leiomyosarcoma. Other characteristics of the tumour which confirmed the diagnosis include nuclear pleomorphism, raised mitotic activity, and areas of necrosis. He had metastatic work-up which showed no evidence of metastatic disease. He was adjudged to be unsuitable for curative surgery in view of the extent of the disease and the associated expected morbidity. He was treated by means of adjuvant chemotherapy which included ifosfamide and this was followed by external beam radiotherapy. Upon completion of the chemotherapy and radiotherapy treatments the patient's symptoms had resolved. At his six-month follow-up, he was asymptomatic in that he did not have any haematuria and his lower urinary tract symptoms had resolved. He had a CT scan which showed significant reduction in the size of the prostatic mass and minimal periprostatic stranding as well as normal looking urinary bladder. His serum PSA was normal. Talapatra et al. [31] stated the following: Limon et al. [16] had studied the cytogenetic analysis of primary leiomyosarcoma of the prostate and reported that their study had revealed clonal chromosomal rearrangement involving Chromosomes 2, 3, 9, 11, and 19; Cambronero et al. [42] reported a case of leiomyosarcoma of prostate which presented as an exophytic tumour mass in the rectum as a rare presentation of leiomyosarcoma of the prostate gland; Cuesta Alcaca et al. [43] reported leiomyosarcoma of the prostate gland which was detected in a patient who underwent TURP for lower urinary tract symptoms diagnosed as benign prostatic hypertrophy, but histological examination of the specimen had revealed leiomyosarcoma of the prostate gland; Chen et al. [44] had stated that rectal examination in leiomyosarcoma of the prostate gland generally tends to reveal a prostatic mass; however, biopsy of the prostatic mass is required for histological examination to confirm the diagnosis; Ahlering et al. [35] reported 11 patients who had leiomyosarcoma; of these 11 patients, 7 had leiomyosarcoma of the urinary bladder, and 4 patients had leiomyosarcoma of the prostate gland. They reported that the patients who did not have bulky tumours underwent surgical resection and they were observed as to if their operative surgical margins and lymph nodes were negative for tumour. The patients who were found to have surgical margins or lymph node positive for tumour received adjuvant external beam radiotherapy and chemotherapy. With regard to the patients who had bulky tumours, they received preoperative chemotherapy with or without radiotherapy which was followed by exenteration. With regard to the outcome, Ahlering et al. [35] reported that, out of the 11 patients, 9 patients did not have any evidence of disease after a mean follow-up of 61 months and the follow-up had ranged between 35 months and 96 months; Camuzzi et al. [45] had reported a patient with leiomyosarcoma of the prostate gland who was successfully treated by means of transperineal radon seed implantation and external beam radiotherapy; Kuroda et al. [46] had reported a case of leiomyosarcoma of the prostate gland which was accompanied by multiple hepatocellular carcinomas who had received combination chemotherapy that consisted of cyclophosphamide, vincristine, Adriamycin, and DTIC (CYDAVIC). He died one year and two months after his initial diagnosis as a result of liver failure. During postmortem examination it was revealed that the histology of the liver tumours was hepatocellular carcinoma and even though the leiomyosarcoma of the prostate gland had invaded the wall of the urinary bladder and the rectum, there was no obvious distant metastasis from the leiomyosarcoma of the prostate gland; Tazi et al. [47] stated the following: a number of treatment modalities had been adopted including radical surgery, radiotherapy, and chemotherapy for the treatment of primary leiomyosarcoma of the prostate gland, but in their opinion a successful outcome had not been achieved in any instance. Leiomyosarcoma of the prostate gland has a poor prognosis, even though the survival time is variable. In view of the aforementioned reasons, it is very important that leiomyosarcoma of the prostate gland is correctly identified and that occurrence of each case of the disease, type of treatment given, and response to treatment should be reported in order to enable the understanding of the natural history of the disease.

Russo et al. [34] reported a 57-year-old man who had a high-grade leiomyosarcoma of the prostate. MRI scan was used to define the extent of the tumour. He was treated by means of chemotherapy and he had a further MRI scan which had shown that the size of the tumour had reduced by 60% and based upon the MRI scan findings the tumour was adjudged to be clinically resectable. He underwent radical cystoprostatectomy but subsequently died of metastatic disease.

Dundore et al. [33] reviewed the notes and pathological findings of 21 cases of carcinosarcoma of the prostate in the Mayo Clinic and reported the results as follows: the mean age of the patients was 68 years and the ages had ranged between 50 years and 89 years; ten of the patients (48%) were previously diagnosed as having acinar adenocarcinoma of the prostate gland two to seventy-three months prior to the diagnosis of carcinosarcoma and the mean time of the diagnosis of acinar adenocarcinoma prior to the diagnosis of carcinosarcoma was 33 months. Eight of the patients, meanwhile, had received androgen deprivation treatment and/or radiotherapy; the Gleason scores of the adenocarcinomas had ranged from Gleason 7 to Gleason 10 and the mean Gleason score was 9 and, furthermore, the median Gleason score was also 9; the various types of sarcomas which were diagnosed were as follows: 13 cases of osteosarcoma, 5 cases of leiomyosarcoma, 1 case of fibrosarcoma, 1 case of malignant fibrous histiocytoma, and 1 case of rhabdomyosarcoma. The grades of the sarcoma components of the tumours had ranged from 2 to 4 and the mean grade was 4. The median grade of the sarcomatous components of the tumour was 4; with regard to the dominant component of the tumours, adenocarcinoma was the dominant histological pattern in 7 cases and sarcoma was the dominant component in 14 cases; with regard to the immunohistochemistry of the tumours, positive immunohistochemical staining cytoplasmic reactivity was revealed for prostate specific antigen or keratin in 16 of 16 cases in the adenocarcinoma component. The sarcoma component exhibited positive staining: in 16 out of 16 cases for vimentin, in 8 out of 16 cases of which 2 were focally positive, in 2 out of 16 cases for S-100 protein, and in 1 out of 16 for desmin; at the time of the initial diagnosis of carcinosarcoma, 11 of the patients were found to have had metastases and of these patients 4 had had metastatic adenocarcinoma prior to the diagnosis of adenocarcinoma; the patients had undergone various types of treatment and they had found that nonsurgical treatment was ineffective; the sites of the metastatic lesions included the lung in 10 cases, bone in 7 cases, brain in 4 cases, liver in 1 case, and the peritoneum in 1 case; with regard to the follow-up of patients, this had ranged between 1 month and 107 months pursuant to the diagnosis of carcinosarcoma and the mean follow-up was 34 months, but the median follow-up was 10 months; with regard to outcome, the mean time to progression of tumour was 23 months, but this had ranged between 1 month and 96 months and the median time to progression was 18 months. Eighteen of the patients had died of carcinosarcoma from 2 months to 107 months following their diagnosis, but the mean and median times of their deaths pursuant to their diagnosis were 34 months and 9.5 months, respectively. The 5-year survival was 41% and the 7-year survival was 14%. The progression and survival of the patients were observed not to be affected by the histological pattern of the carcinosarcomas. Dundore et al. [33] concluded that carcinosarcoma of the prostate gland is an aggressive malignancy, irrespective of the histological type of the carcinosarcoma of the prostate gland.

Globally, a large number of patients undergo treatment for adenocarcinoma of the prostate gland with curative intent in the form of radiotherapy (external beam radiotherapy or brachytherapy) plus or minus adjuvant hormonal therapy and out of these patients a number of patients subsequently develop relapse or progress of their tumours. What is not known globally is the percentage of patients who were initially diagnosed as having adenocarcinoma of prostate who subsequently develop either de novo carcinosarcoma including leiomyosarcoma of the prostate or dedifferentiated carcinoma of the prostate gland including leiomyosarcoma of the prostate. Perhaps the reason behind the lack of information regarding the percentage of patients globally who develop carcinosarcoma alone or in association with adenocarcinoma may be due to the fact that globally routine rebiopsies are not generally undertaken in the majority of patients who are treated globally for adenocarcinoma of prostate gland in the form of radiotherapy plus or minus hormonal therapy. It would be argued perhaps if all patients who develop relapse after radiotherapy/hormonal treatment for adenocarcinoma of the prostate gland undergo rebiopsies of the prostate a number of cases of dedifferentiated carcinomas would be found alone or in association with adenocarcinoma. In view of the finding that carcinosarcomas have been found to be aggressive tumours that would require aggressive treatment with curative intent in the form multimodal treatment including radical surgery, chemotherapy, and radiotherapy in cases of patients who are fit to undergo aggressive treatment, it would be argued that a global multicentre study should be undertaken in which patients whose adenocarcinomas have relapsed given the chance to undergo rebiopsies of their prostates to see if their carcinomas remain the same or they have subsequently developed different types of prostate cancers. If a pilot study shows a high percentage of patients developing other types of prostate cancers, then perhaps new guidelines could be formulated globally for the follow-up of patients with adenocarcinoma of the prostate. Finally, it is conjectural, but it may be that the percentage of other types of carcinomas of the prostate that are reported globally is higher than is believed and this would hopefully be known in the future if lots of patients undergo rebiopsies of their prostatic lesions for histological examination.

Lida et al. [38] in 1998 reported two patients with PLSOPs. The first patient was a 45-year-old man who presented with lower urinary tract symptoms. He had ultrasound scan and CT scan which revealed a tumour in his prostate gland. He had ultrasound-guided biopsy of the prostatic lesion and histological examination of the specimen revealed leiomyosarcoma of the prostate gland. He underwent radical cystoprostatectomy. He was alive at the time of publication of the paper 12 months after his operation. The second patient was a 63-year-old man who was admitted for the treatment of lung and colon cancers. He had CT scan which had shown multiple tumours in the lung, in the liver, and bilaterally in the kidney as well as prostate gland. Histological examination of biopsies from the prostate gland had confirmed the presence of leiomyosarcoma of the prostate. Lida et al. [38] also reviewed 57 cases of primary leiomyosarcoma of the prostate gland which had been reported in the Japanese literature.

Palma et al. [39] reported a case of leiomyosarcoma of the prostate gland in association with an incidental adenocarcinoma of the prostate gland in 1983 and in 1997 Stallwood and Davidson [40] reported leiomyosarcoma of the rectum and prostate gland.

Mansouri et al. [48] reported a 14-year-old boy who presented with perineal pain on voiding and during defecation as well as dysuria and haematuria. His rectal examination revealed an indurated left lobe of the prostate gland. He had intravenous urography and CT scan of abdomen and pelvis which showed the urinary bladder displaced anteriorly compressed by a large pelvic mass, 15 cm × 11 cm, multiseptated pelvic mass with thickened irregular walls which had displaced the urinary bladder and rectum. He had surgical biopsy of the mass elsewhere and histological examination of the specimen had shown a smooth muscle neoplasm which did not have any features that would exclude leiomyosarcoma. He underwent radical cystoprostatectomy and pelvic lymph node dissection. The radical prostatectomy specimen contained a tumour on the left side which was not circumscribed and the tumour had extended from the apex to the base. Histological examination of the tumour had shown an infiltrative tumour that had invaded and displaced the normal prostate stroma of glands and these tumours had abnormal mitotic figures and giant cells. Necrosis was observed and a high mitotic count of 14 per 10 high-power fields was also observed. Immunohistochemical staining of the tumour revealed strong cytoplasmic positive staining for actin, desmin, and vimentin, but there was negative staining for keratin and S-100 protein. The surgical margin at the resection site was involved by tumour as well as extraprostatic extension. Three left iliac nodes contained metastatic tumour. He was treated by means of adjuvant radiotherapy and received 46 Gy of therapy, but he died as a result of metastases in the lung, liver, and bone as well as gross local recurrence 4 months following his initial diagnosis.

All the aforementioned information could be confusing to a clinician who is faced with a patient who is diagnosed with PLSOP. One of the questions that would be on the mind of the clinician is what is the best treatment option for the disease (PLSOP) Sexton et al. [32] had recommended that multimodality treatment include surgery, radiotherapy, and chemotherapy. Some authors [2, 11, 26, 32] had iterated that the overall outcome for PLSOPs is not good, and 50% to 75% of patients tend to die of their leiomyosarcoma between 3 months and 72 months. Sexton et al. [32] stated that the outcome of PLSOPs tends to improve in patients who do not have evidence of distant metastasis at initial manifestation and in those patients who have localized disease in whom complete resection of the tumour can be undertaken with histological evidence of negative surgical margins.

## 4. Conclusions

Leiomyosarcoma of the prostate gland is a rare tumour which can afflict children and adults. On the whole the prognosis is poor. Surgery with or without chemotherapy/radiotherapy would appear to be the mainstay of treatment for leiomyosarcoma of the prostate for operable cases, but generally there is no consensus opinion on the best therapeutic approach. Most cases of PLSOP at the time of initial presentation tend to be diagnosed in an advanced stage of the disease. There is the need for all cases of PLSOP diagnosed to be entered into a global multicentre trial in order to find therapeutic modalities that would improve the prognosis of this aggressive disease. Perhaps if all patients who have developed progressive disease or relapse of local disease in cases of adenocarcinoma prostate undergo repeat prostate biopsies early, some cases of dedifferentiated leiomyosarcomas of the prostate may be diagnosed early.

## Figures and Tables

**Table 1:** List of some of the reported cases of primary leiomyosarcoma of the prostate gland.

Authors and reference	Year of publication	Number of cases
Dotan et al. [30]	2006	8
Talapatra et al. [31]	2006	1
Sexton et al. [32]	2001	12
Cheville et al. [2]	1995	23
Dundore et al. [33]	1995	5
Russo et al. [34]	1993	1
Ahlering et al. [35]	1988	4
Vandoros et al. [13]	2008	1
Singh et al. [10]	2013	1
Dubey et al. [11]	2010	1
Chen et al. [15]	2000	1
Vakilha et al. [29]	2004	1
Horiguchi et al. [28]	2014	1
Sastri et al. [14]	2002	1
Germiyanoglu et al. [9]	1994	1
Barone and Joelson [36]	1950	1
Yee et al. [37]	2009	1
Lida et al. [38]	1998	2 cases, but they also reviewed 57 cases in the Japanese literature previously published
Palma et al. [39]	1983	1
Stallwoodand Davidson [40]	1977	1
Moreira et al. [41]	2004	1
Limon et al. [16]	1986	1
Cambronero et al. [42]	1999	1
Cuesta Alcaca et al. [43]	2000	1
Chen et al. [44]	2005	1
Camuzzi et al. [45]	1981	1
Kuroda et al. [46]	1994	1
Tazi et al. [47]	2001	2
Mansouri et al. [48]	2001	1 (14-year-old boy)
Mondaini et al. [49]	2005	3
